# Transcription factor WRKY22 regulates canker susceptibility in sweet orange (*Citrus sinensis* Osbeck) by enhancing cell enlargement and *CsLOB1* expression

**DOI:** 10.1038/s41438-021-00486-2

**Published:** 2021-03-01

**Authors:** Qin Long, Meixia Du, Junhong Long, Yu Xie, Jingyun Zhang, Lanzhen Xu, Yongrui He, Qiang Li, Shanchun Chen, Xiuping Zou

**Affiliations:** Citrus Research Institute, Southwest University/Chinese Academy of Agricultural Sciences, Chongqing, People’s Republic of China

**Keywords:** Biotic, Plant molecular biology

## Abstract

Pathological hypertrophy (cell enlargement) plays an important role in the development of citrus canker, but its regulators are largely unknown. Although WRKY22 is known to be involved in pathogen-triggered immunity and positively regulates resistance to bacterial pathogens in Arabidopsis, rice and pepper, the CRISPR/Cas9-mediated partial knockout of *CsWRKY22* improves resistance to *Xanthomonas citri* subsp. *citri* (Xcc) in Wanjincheng orange (*Citrus sinensis* Osbeck). Here, we demonstrate that CsWRKY22 is a nucleus-localized transcriptional activator. *CsWRKY22*-overexpressing plants exhibited dwarf phenotypes that had wrinkled and thickened leaves and were more sensitive to Xcc, whereas *CsWRKY22*-silenced plants showed no visible phenotype changes and were more resistant to Xcc. Microscopic observations revealed that the overexpression of *CsWRKY22* increased cell size in the spongy mesophyll. Transcriptome analysis showed that cell growth-related pathways, such as the auxin and brassinosteroid hormonal signaling and cell wall organization and biogenesis pathways, were significantly upregulated upon *CsWRKY22* overexpression. Interestingly, CsWRKY22 activated the expression of *CsLOB1*, which is a key gene regulating susceptibility to citrus canker. We further confirmed that CsWRKY22 bound directly to the W-boxes just upstream of the transcription start site of *CsLOB1* in vivo and in vitro. We conclude that CsWRKY22 enhances susceptibility to citrus canker by promoting host hypertrophy and *CsLOB1* expression. Thus, our study provides new insights into the mechanism regulating pathological hypertrophy and the function of WRKY22 in citrus.

## Introduction

Citrus canker, induced by *Xanthomonas citri* subsp. *citri* (Xcc), is one of the most serious diseases facing the global citrus industry^1,2^. The key pathogenic factors and the molecular mechanisms of the host response to Xcc are not well understood^3–5^, which severely limits the progress of citrus disease resistance breeding. The diagnostic symptoms for citrus canker disease are hypertrophy (cell enlargement), hyperplasia (cell overdivision) and necrosis (cell death)^3^. Pathological hypertrophy and hyperplasia of host cells are the primary conditions for pustule formation and consequent canker symptoms and the spread of pathogens on the plant surface^3^. The inhibition or disruption of pathological hypertrophy and hyperplasia can efficiently repress pustule formation and pathogen spread and even confer resistance to citrus canker^6,7^; therefore, these are potential strategies for the efficient management of citrus canker. Thus, understanding the molecular mechanisms involved in pathological hypertrophy and hyperplasia in citrus could stimulate renewed efforts to develop more effective and economical citrus canker control methods.

Pathogenic bacteria of the genus *Xanthomonas* secrete transcription activator-like effector (TALE) proteins into plant cells to manipulate the expression of host genes, which results in host hypertrophy, hyperplasia, and subsequently complex disease phenotypes. The TALE AvrBs3 from *X. campestris* pv. *vesicatoria* activates the expression of the bHLH transcription factor UPA, thus inducing hypertrophy in pepper and tobacco^8,9^. PthA effectors from Xcc target different susceptibility genes, including *CsLOB1*, *CsDiox*, *CsCYP*, and *CsMAF1*, to promote hypertrophy and/or hyperplasia in citrus^10,11^. CsLOB1 (lateral organ boundary 1), a plant-specific transcription factor in the LBD (lateral organ boundary domain) family, plays a key role in symptom formation and the bacterial growth of canker in citrus^11–13^. Furthermore, the effector PthA4 secreted by the canker pathogen directly binds to the effector binding element in the *CsLOB1* promoter to upregulate the expression of *CsLOB1*^11^. Pathogen effector-elicited hypertrophy (enlargement or lengthening) of mesophyll cells also contributes to pathogen dispersal and feeding^14^. In citrus, how the Xcc pathogen regulates host hypertrophy to promote canker development and bacterial spread is still to be understood.

WRKY transcription factors that contain a well-conserved WRKY (WRKYGQK) domain and a C2H2 or C2HC zinc-finger motif constitute a large family in plants and participate in the regulation of plant growth, development, metabolism, and multiple responses to abiotic and biotic stresses^15,16^. WRKY22 can be activated by pathogen-associated molecular patterns (PAMPs), including flagellin and chitin, which can induce the mitogen-activated protein kinase (MAPK) cascade, and is considered a marker gene of pathogen-triggered immunity (PTI)^17,18^. In Arabidopsis, rice and pepper, the expression of *WRKY22* is upregulated by bacterial pathogens and contributes to host resistance^19–21^. However, in our previous works, *CsWRKY22* was induced by Xcc in a susceptible citrus variety, and the CRISPR/Cas9-mediated partial knockout of *CsWRKY22* increased resistance to Xcc in Wanjincheng orange (*Citrus sinensis* Osbeck), indicating that *CsWRKY22* functions as a disease susceptibility gene for citrus canker^22^. The mechanism underlying CsWRKY22-mediated canker susceptibility remains to be elucidated.

In the present study, transgenic Wanjincheng orange with overexpression and RNAi knockdown of *CsWRKY22* was generated. The phenotypes and canker resistance of the transgenic plants were investigated, and RNA sequencing (RNA-Seq) was carried out to assess its downstream transcriptional effects. Moreover, dual LUC assays, yeast one-hybrid (Y1H) assays, electrophoretic mobility shift assays (EMSAs) and transient gene expression analysis were used to characterize the regulation of *CsLOB1* by CsWRKY22. We found that CsWRKY22 promotes host hypertrophy and *CsLOB1* expression, thus increasing host susceptibility to citrus canker.

## Results

### CsWRKY22 is a nucleic protein with transcriptional activation activity

**Fig. 1:**
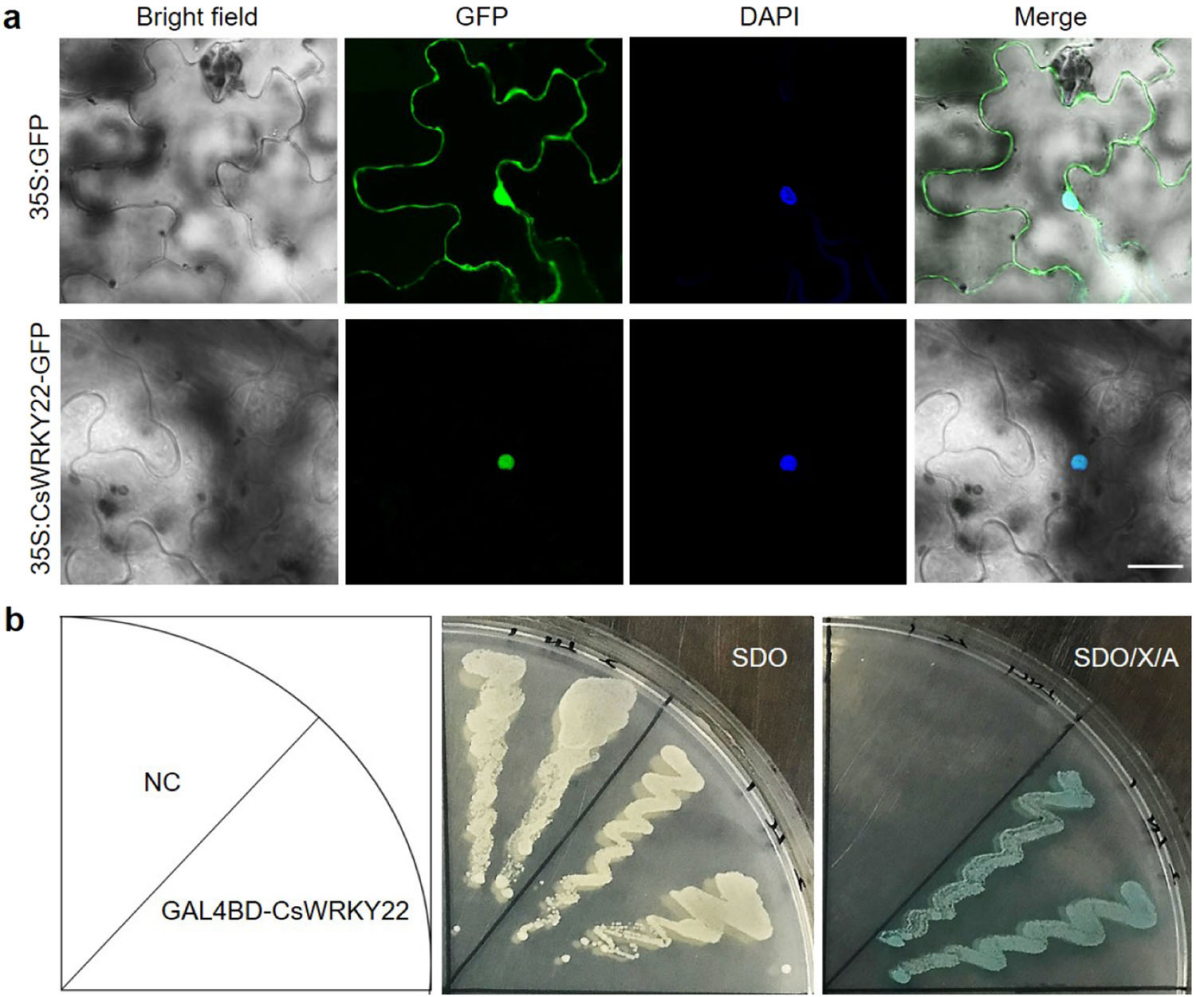
Subcellular localization and transcriptional activity assays of CsWRKY22. **a** CsWRKY22 is localized in the nucleus based on transient expression in tobacco leaves. The nucleus was stained with DAPI (blue). Bar = 30 μm; **b** Transcriptional activation of CsWRKY22 in yeast. Yeast strain Y2HGold carrying GAL4BD-CsWRKY22 and pGBKT7 empty vector (NC) were cultured on SD/-Trp (SDO) or SDO medium supplemented with X-α-gal and AbA (SDO/X/A) at 30 °C for 3 d

To probe the subcellular localization properties of CsWRKY22, a vector containing a fusion between the CsWRKY22 coding sequence and the GFP gene under the control of the CaMV (cauliflower mosaic virus) 35S promoter and a control vector including the *35S:GFP* cassette were constructed. Both the fusion and control vectors were transiently introduced into the tobacco leaves. Microscopic observation showed that the GFP signal of the control was detected in both the nucleus and cytoplasm, while the green fluorescence signal of the CsWRKY22-GFP fusion was observed exclusively in the nuclei (Fig. 1a), indicating that CsWRKY22 is a nuclear protein.

To determine whether CsWRKY22 has a transcriptional activating role, the full-length *CsWRKY22* was fused downstream to the GAL4 DNA binding domain (GAL4BD) in the pGBKT7 vector and transformed into Y2HGold yeast. All yeast cells showed normal growth on the synthetic dropout medium without tryptophan (SDO), whereas only the cells transformed with GAL4BD-CsWRKY22 vectors survived and turned blue when they were cultured on a selective medium supplemented with X-α-gal and aureobasidin A (SDO/X/A) (Fig. 1b). These results suggest that the GAL4BD-CsWRKY22 fusion protein was able to activate the expression of the reporter genes *MEL1* and *AUR1*, indicating that CsWRKY22 has transcriptional activation potential.

### Overexpression of *CsWRKY22* affects plant phenotype and cell size

**Fig. 2:**
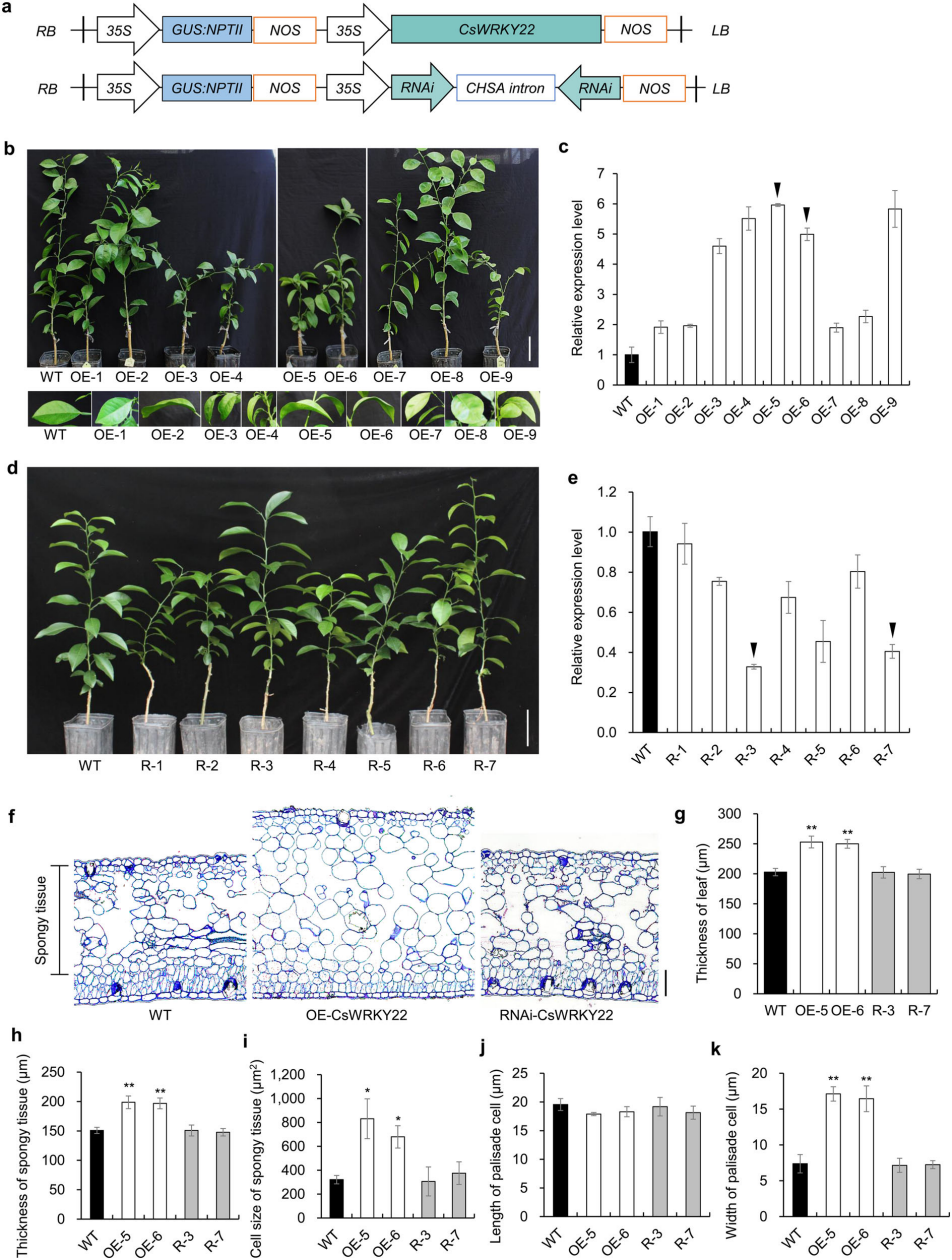
Phenotypic characteristics of *CsWRKY22* transgenic plants. **a** Recombinant plasmids used for overexpression and RNAi assays. 35S, CaMV35S promoter; GUS::NPTII *GUS::NPTII* fusion gene, NOS NOS terminator, RB right border, LB left border; **b** Phenotypes of one-year-old transgenic plants overexpressing *CsWRKY22* and of the WT control. Bar = 10 cm; **c** *CsWRKY22* expression in transgenic plants overexpressing *CsWRKY22* and WT assessed by qRT-PCR; **d** Phenotypes of 10-month-old *CsWRKY22*-silenced and WT plants. Bar = 10 cm; **e** *CsWRKY22* expression in *CsWRKY22*-silenced plants and WT control; **f** Microscopic observation of leaf cellular morphology. Bar = 30 μm; **g**–**k** Statistical analyses of the leaf thickness (**g**), spongy tissue thickness (**h**), spongy tissue cell size (**i**), palisade cell length (**j**), and width (**k**) in transgenic plants and the WT control. All the data are given as the means ± SDs (*n* = 3). Arrows indicate the representative lines that were used in further investigations. Nine leaves of each line were used for statistical analysis. Asterisks indicate significant differences between transgenic plants and the WT control by Student’s *t*-test (*P < 0.05; **P < 0.01). WT, wild type; OE-1~9, *CsWRKY22-*overexpressing lines; R-1~7, *CsWRKY22*-silenced lines.

To further investigate the role of Cs*WRKY22* in citrus, we generated nine overexpression (OE) and seven RNA interference (RNAi) (Fig. 2a) transgenic Wanjincheng orange plants *via Agrobacterium*-mediated transformation. The OE-3, OE-4, OE-5, OE-6, and OE-9 lines, with relatively high expression levels of Cs*WRKY22*, showed dwarf phenotypes with wrinkled and thickened leaves compared with those of wild-type (WT) plants (Fig. 2b, c and Fig. S1), whereas no visible changes were observed in the RNAi transgenic plants (Fig. 2d). Based on the expression level of Cs*WRKY22*, we selected two lines as representatives (Fig. 2c, e). Further microscopic observation and statistical data analysis showed that the spongy parenchyma cells in the *CsWRKY22*-overexpressing plants were significantly enlarged compared with those in the WT plants, and the spongy cells in the *CsWRKY22*-overexpressing plants were 2–3 times the size of those in the WT control. The palisade cells of *CsWRKY22*-overexpressing leaves also became wider than those of the WT. No differences in cell morphology were detected between the RNAi and WT plants (Fig. 2f–k). These results indicated that the overexpression of *CsWRKY22* could increase cell size in citrus.

### CsWRKY22 increases susceptibility to *Xanthomonas citri* subsp. *citri*

**Fig. 3:**
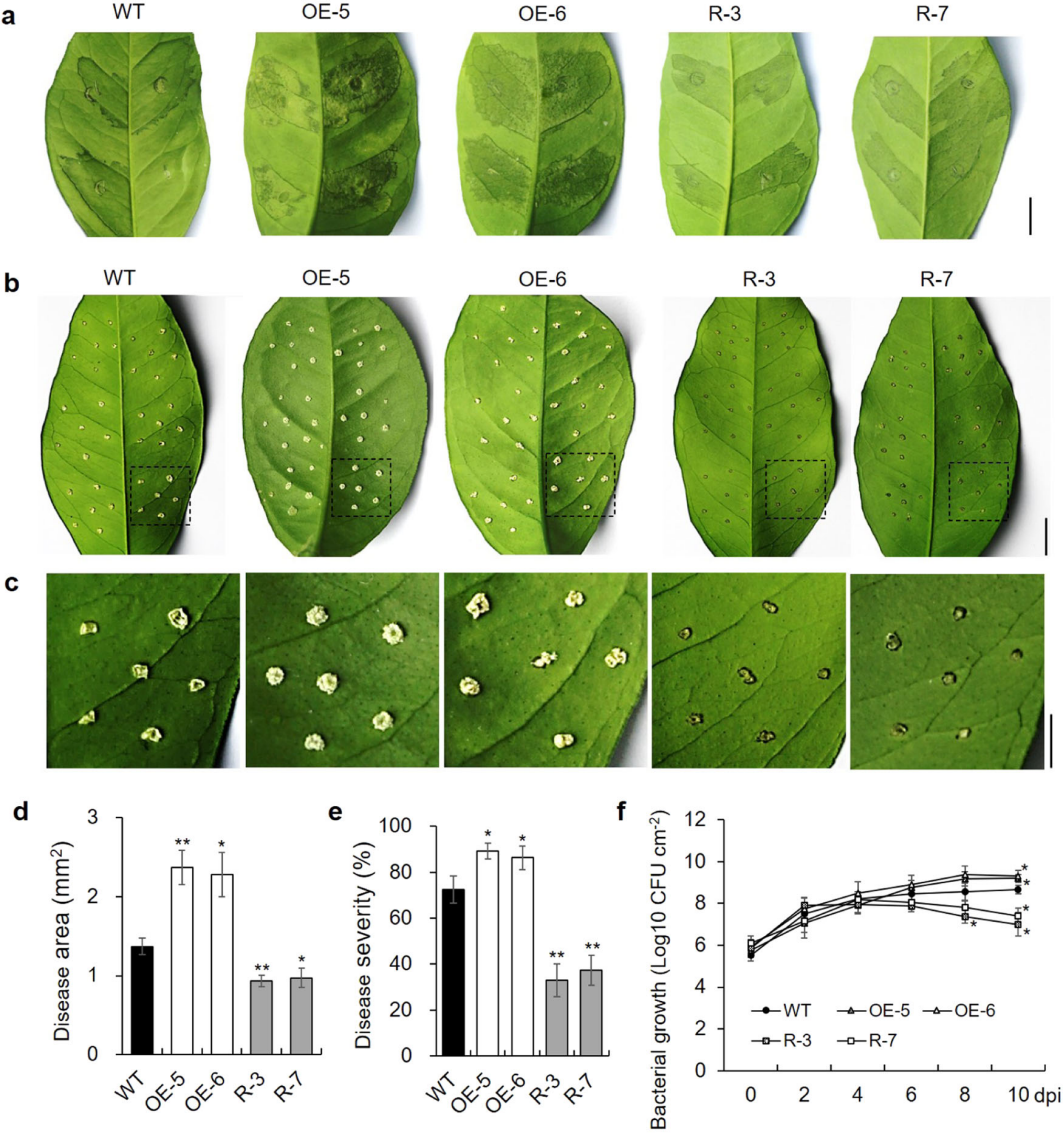
Evaluation of resistance to citrus canker in transgenic plants and the WT control. **a** Canker symptoms on leaves after in vitro inoculation with Xcc by the infiltration method. Scale bar = 1 cm; **b** Canker symptoms on leaves after inoculation with Xcc by the pinprick inoculation method. Scale bars = 1 cm; **c** Magnified images of the original photograph in **b**. Scale bar = 0.5 cm; **d**–**f** Statistical analyses of the disease area (**d**), disease severity (**e**), and bacterial growth (**f**) in leaves after inoculation with Xcc. Fully expanded leaves were inoculated with 1 × 10^5^ CFU/mL Xcc. Photographs were taken at 10 dai. Data represent the averages ± SDs from three biological repeats, and nine independent leaves were observed for each experiment. Asterisks indicate significant differences between transgenic plants and the WT by Student’s *t*-test (*P < 0.05; **P < 0.01). WT, wild type; OE-5 and OE-6, *CsWRKY22-*overexpressing lines; R-3 and R-7, *CsWRKY22*-silenced lines; dpi, days post inoculation

To evaluate canker resistance in the *CsWRKY22* transgenic plants, leaves of transgenic and WT plants were inoculated with an Xcc suspension by the infiltration method^23^. Ten days after inoculation (dai), *CsWRKY22*-overexpressing plants showed much more serious symptoms than WT plants, whereas the opposite trend was observed in RNAi plants (Fig. 3a). The pinprick inoculation method was used to quantitatively evaluate disease resistance in the plants. At 10 dai, leaves of *CsWRKY22*-overexpressing plants exhibited larger pustule eruptions, while the RNAi plant leaves showed significantly alleviated symptoms compared with the leaves of WT (Fig. 3b, c). The statistical analysis showed that the disease area increased by 72.7% (OE-5) and 66.4% (OE-6) in the overexpressing plants but decreased by 31.9% (R-3) and 29.0% (R-7) in the RNAi plants compared to that in the WT control (Fig. 3d). The disease severity was 72.5% in WT, increased to 89.2% (OE-5) and 86.3% (OE-6) in the overexpressing plants, and decreased to 32.9% (R-3) and 37.3% (R-7) in the RNAi plants (Fig. 3e). Furthermore, there were significantly more colony-forming units (CFUs) in the overexpressing plants but significantly fewer CFUs in the RNAi plants than in the WT at 10 dai (Fig. 3f). These results confirm that *CsWRKY22* positively regulates susceptibility to citrus canker in Wanjincheng orange.

### *CsWRKY22* overexpression leads to extensive transcriptional reprogramming of genes related to cell enlargement

**Fig. 4:**
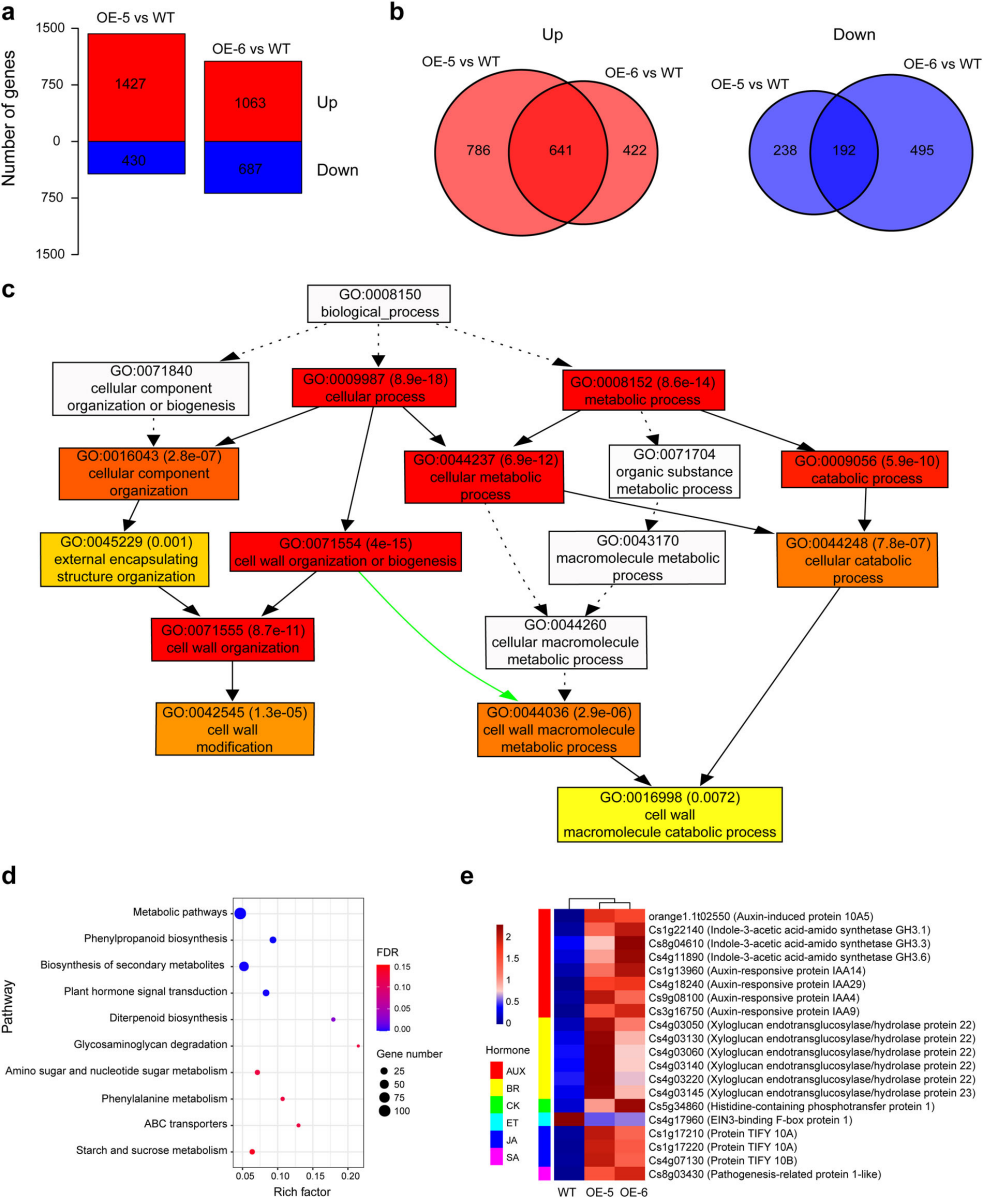
Transcriptome analysis of *CsWRKY22*-overexpressing and WT plants. **a** The number of differentially expressed genes (DEGs) between *CsWRKY22*-overexpressing and WT plants (red bars: upregulated, blue bars: downregulated); **b** overlap in upregulated genes and downregulated genes; **c** Thumbnail view of the directed acyclic graph for the cell wall; **d** KEGG pathways enriched among the DEGs; **e** Expression of hormone-related genes in *CsWRKY22*-overexpressing and WT plants. WT wild type; OE-5 and OE-6, *CsWRKY22-*overexpressing lines, FDR false discovery rate, AUX auxin, BR brassinolide, CK cytokinin, ET ethylene, JA jasmonic acid, SA salicylic acid.

To explore the molecular mechanism of the involvement of Cs*WRKY22* in plant growth and resistance to citrus canker and to identify potential target genes that may be regulated by CsWRKY22, mRNA sequencing (RNA-seq) analysis was performed on *CsWRKY22-*overexpressing and WT plants. After filtering, approximately 50 million clean reads were scored in each sample (Table S1). A total of 1857 and 1750 genes showed altered transcript levels (fold change ≥ 2, FDR < 0.05) in the OE-5 and OE-6 lines compared with the WT, respectively. There were 1427 upregulated and 430 downregulated genes in the OE-5 line and 1063 upregulated and 687 downregulated genes in the OE-6 line (Fig. 4a and Table S2). A total of 833 differentially expressed genes (DEGs) were shared by the two lines (Fig. 4b). Then, GO (Gene Ontology) and KEGG (The Kyoto Encyclopedia of Genes and Genomes) pathway enrichment analyses of the 833 DEGs were performed. The GO results indicated that these DEGs were classified into 132 biological processes (FDR < 0.05) (Table S3). The resultant directed acyclic graphs (DAGs) showed that GO terms associated with development, cell wall, response to stimulus (including response to stress and response to hormone), and kinase were overrepresented (Figs. S2 and Fig. 4c). Specifically, the majority of the 29 DEGs of GO:0071554 (cell wall organization and biogenesis) term, such as expansin, β-1,4-endoglucanase, pectin lyase, and cellulose synthase genes, which contribute to cell elongation and enlargement^24–29^, were upregulated in the overexpressing lines (Table S3 and Table 1). Auxin and brassinosteroid (BR) hormones play important roles in plant cell growth^30–33^. Consistent with this, KEGG pathway analysis retrieved the plant hormone signal transduction pathway (cit04075) containing 20 genes (FDR < 0.05) (Fig. 4d and Table S4); in this pathway, the auxin-inducible genes SAUR (auxin-induced protein), GH3 (indole-3-acetic acid-amido synthetase), IAA (auxin-responsive protein), and BR-regulated xyloglucan endotransglucosylase/hydrolase were overrepresented (Fig. 4e and Fig. S3). These results show that WRKY22 promotes cell growth through the activation of auxin and BR hormonal signaling pathways as well as cell wall organization and biogenesis processes.

**Table 1:** Expression of cell wall-related genes in *CsWRKY22*-overexpressing plants

Gene ID	Name	Fold change	
		OE-5	OE-6
Cs2g17090	β-1,4-endoglucanase	5.59	7.32
Cs2g12180	β-1,4-endoglucanase	5.06	2.02
orange1.1t02681	β-xylosidase	11.54	3.23
Cs7g17340	Callose synthase	2.62	2.23
Cs4g01990	Cellulose synthase	18.10	7.73
Cs2g04590	Cellulose synthase-like protein	154.88	52.67
Cs8g01880	Expansin	12.27	3.86
orange1.1t00255	Fasciclin-like arabinogalactan protein	5.05	5.04
Cs7g07900	Galacturonosyltransferase-like	2.39	2.33
Cs7g18460	Glucomannan 4-β-mannosyltransferase 2	7.18	11.19
Cs3g24380	Glycosyl transferase	20.17	11.63
Cs2g10240	Patellin	51.16	22.19
Cs7g21940	Pectate lyase	2.16	2.85
Cs8g11330	Pectate lyase	9.22	8.17
Cs1g16560	Pectinesterase	8.50	4.17
Cs1g16550	Pectinesterase	4.41	12.52
orange1.1t02719	Pectinesterase	5.96	2.77
Cs2g07660	Pectinesterase-like	5.43	3.95
Cs5g03170	Polygalacturonase	4.02	8.74
Cs9g19250	Polygalacturonase	0.24	0.11
Cs1g19290	Serine/threonine-protein kinase	4.97	3.24
Cs6g19640	UDP-arabinopyranose mutase	2.61	3.51
orange1.1t01956	UDP-glucose 4-epimerase	3.30	3.60
Cs6g02710	Wall-associated receptor kinase-like	3.21	2.90
Cs3g07330	Cytochrome P450	6.89	14.27
Cs3g25780	Cytochrome P450	2.05	2.77
Cs5g25070	Cytochrome P450	437.76	413.86
Cs5g25060	Cytochrome P450	6.69	9.36
Cs7g21900	Uncharacterized protein	0.33	0.20

OE-5 and OE-6, transgenic lines overexpressing *CsWRKY22*.

### CsWRKY22 activates *CsLOB1* expression by directly binding to a specific promoter sequence

**Fig. 5:**
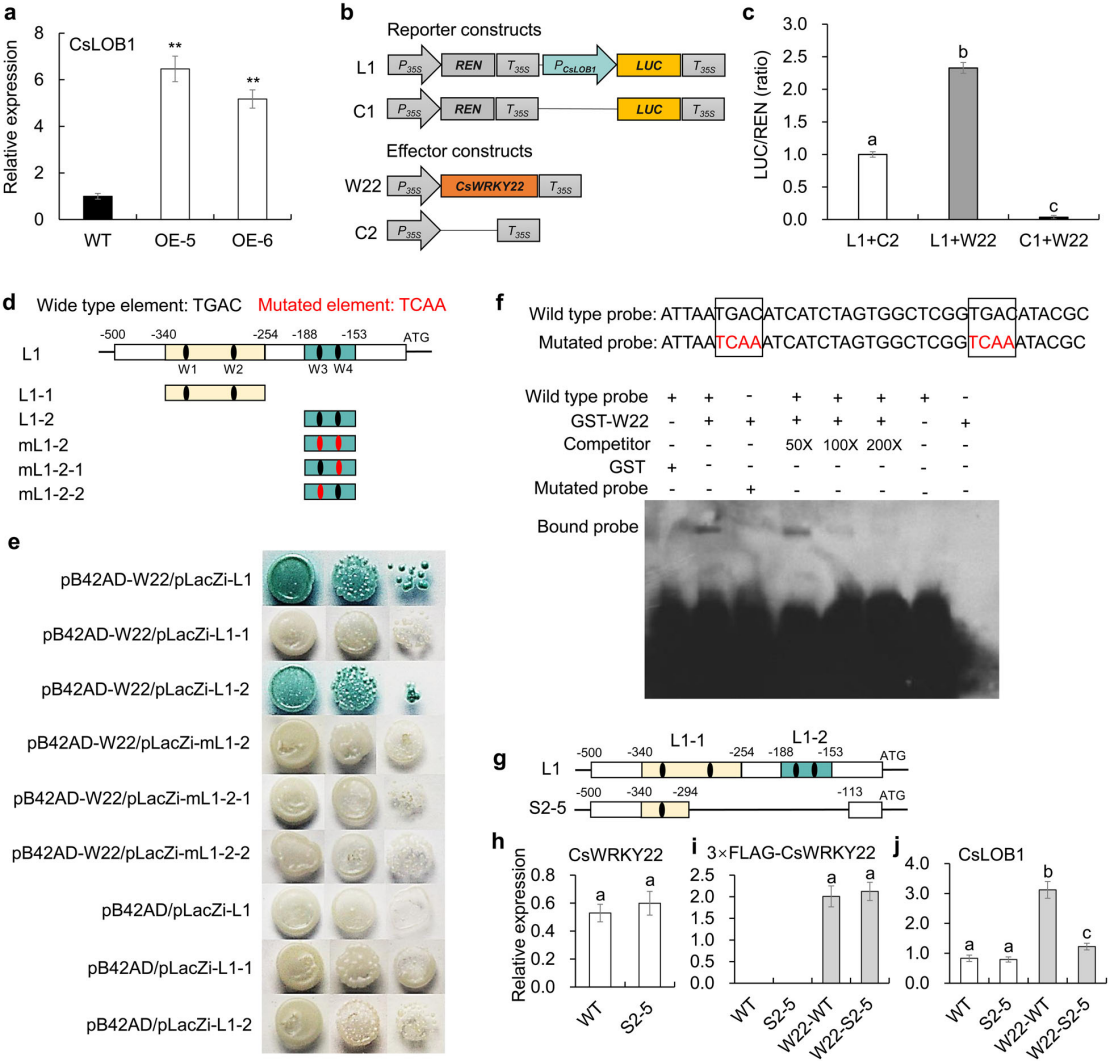
CsWRKY22 directly binds to and activates the promoter of *CsLOB1*. **a** The transcription level of *CsLOB1* in *CsWRKY22*-overexpressing plants (OE) and the wild-type (WT) control. **b** Reporter and effector vectors constructed for dual LUC assays. The *CsLOB1* promoter was inserted ahead of the LUC gene to form a reporter construct (L1), and the empty vector was used as a control (C1). CaMV35S promoter-driven CsWRKY22 (W22) was used as the effector, and the empty vector was used as the control (C2). LUC firefly luciferase, REN Renilla luciferase, P_35S_ CaMV35S promoter, T_35S_ CaMV35S terminator. **c** Transient expression assay of promoter activity in tobacco leaves, shown as ratios of LUC to REN; **d** Schematic diagrams of *CsLOB1* promoter fragments used for the Y1H assay. The black dots represent the original W-boxes. The red dots represent mutated W-boxes; **e** Y1H assays of CsWRKY22 and *CsLOB1* promoter fragments; **f** EMSAs of the specific binding of CsWRKY22 to the W-box sequence in the *CsLOB1* promoter. Elements shown in red are mutated W-boxes. The GST-CsWRKY22 protein (GST-W22) was incubated with a probe labeled with biotin, accompanied by or without native competitor DNA. +, presence; −, absence; **g** Schematic representation of the deletion of the S2-5 mutant line reported by Peng et al.^7^; **h** Expression of *CsWRKY22* in the WT and S2-5 mutant lines; **i**, **j** Expression levels of *CsWRKY22* and *CsLOB1* in the WT and S2-5 lines with or without the transient introduction of *3×FLAG*-*CsWRKY22* (W22). All the data are shown as the means ± SDs (*n* = 3). Asterisks indicate significant differences between transgenic plants and the WT control by Student’s *t*-test (**P < 0.01). Different letters above the bars represent significant differences based on Duncan’s multiple range test (*P* < 0.05)

The transcriptome results also showed that the citrus canker susceptibility gene *CsLOB1* (Cs7g27640), which plays a key role in canker symptom formation and bacterial growth^11,12^, was significantly upregulated in *CsWRKY22*-overexpressing lines (Table S2). This result was further confirmed by qPCR (Fig. 5a). We were thus curious to know whether *CsLOB1* is a direct target gene of CsWRKY22. It has been demonstrated that WRKY transcription factors bind to W-boxes (which contain TGAC core sequences) in the promoters of their target genes^34^. We identified four TGAC core sequences in the 500-bp sequence of the *CsLOB1* promoter^7^ (Fig. S4). Thus, we first investigated whether the *CsLOB1* promoter fragment was activated by CsWRKY22 using a dual luciferase (LUC) assay. The *CsLOB1* promoter was inserted ahead of the LUC gene in the pGreenII 0800 vector to form the reporter construct (L1), and the CaMV35S promoter-driven CsWRKY22 (W22) was used as the effector (Fig. 5b). Transient expression analysis in tobacco showed that the LUC/REN ratios in leaves carrying both the W22 effector and the L1 reporter were significantly greater than those in leaves carrying L1 or W22 alone (Fig. 5c). These observations showed that CsWRKY22 could activate the *CsLOB1* promoter.

To demonstrate the binding of CsWRKY22 to the *CsLOB1* promoter, we performed a yeast one-hybrid (Y1H) assay. Two different subfragments of the *CsLOB1* promoter (L1-1, −340 to −254 bp; L1-2, −188 to −153 bp) containing W1-boxes and W2-boxes and W3-boxes and W4-boxes, respectively (Fig. 5d), were individually inserted into the pLacZi vector. The resultant constructs were then separately transformed into a yeast strain expressing CsWRKY22 fused with the activation domain. The pB42AD-CsWRKY22/pLacZi-L1-1 and the negative controls pB42AD/pLacZi-L1, pB42AD/pLacZi-L1-1, and pB42AD/pLacZi-L1-2 did not turn blue on synthetic dropout medium without tryptophan or on uracil (SD/-Trp/-Ura) plates supplemented with X-gal (5-bromo-4-chloro-3-indolyl β-d-galactoside); nevertheless, the pB42AD-CsWRKY22/pLacZi-L1 and pB42AD-CsWRKY22/pLacZi-L1-2 turned blue on SD/-Trp/-Ura/X-gal plates (Fig. 5e), indicating that CsWRKY22 binds directly to the L1-2 region of the *CsLOB1* promoter. To further determine whether CsWRKY22 specifically binds to the W-box regions of the L1-2 region, the mutated (TCAA) W-box was used to generate baits (Fig. 5d). pB42AD-CsWRKY22/pLacZi-mL1-2 (mutated W3-boxes and W4-boxes), pB42AD-CsWRKY22/pLacZi-mL1-2-1 (mutated W4-box), and pB42AD-CsWRKY22/pLacZi-mL1-2-2 (mutated W3-box) did not turn blue on SD/-Trp/-Ura/X-gal plates (Fig. 5e). These results showed that CsWRKY22 regulates *CsLOB1* expression by specifically binding to the two W-boxes near the transcription start site (Fig. S4) and that both W-boxes are indispensable.

We also used an electrophoretic mobility shift assay (EMSA) to determine whether CsWRKY22 specifically binds to the W-box motifs of the *CsLOB1* promoter in vitro. CsWRKY22 was expressed in *Escherichia coli* (BL21) as a fusion protein containing a GST tag, and the GST-CsWRKY22 fusion protein was purified. Then, synthesized probes from −188 to −153 bp of the *CsLOB1* promoter region containing normal or mutant W-boxes were prepared (Fig. 5f). GST protein and the original probe were used as negative controls. Incubating the purified GST-CsWRKY22 protein with the original probe, a retarded band representing the binding of the protein to the probe DNA was detected; this retarded migration was decreased in a dose-dependent manner when the unlabeled competitor was added (Fig. 5f). In contrast, the binding of the GST-CsWRKY22 protein to the DNA probe was prevented when the W-boxes in the probe were mutated (Fig. 5f). These results indicate that the CsWRKY22 protein binds directly and specifically to the W-box elements in the L1-2 region of the *CsLOB1* promoter in vitro.

To further verify the transcriptional regulation of *CsLOB1* by CsWRKY22, a 3×FLAG-CsWRKY22 fusion protein under the control of the CaMV35S promoter was transiently introduced into the leaves of the Wanjincheng orange mutant line S2-5^7^ and the WT control plants. The S2–5 line has a deletion of the 180-bp sequence (−294 to −113 bp) of the *CsLOB1* promoter^7^, and the deletion contains only the L1−2 region with the W3-boxes and W4-boxes bound by the CsWRKY22 protein (Fig. 5g). qPCR analysis showed that the basic expression of *CsWRKY22* was comparable between the WT and S2–5 lines (Fig. 5h). *3×FLAG-CsWRKY22* was successfully expressed in the WT and S2–5 lines, and transformants with similar expression levels were selected for further investigation (Fig. 5i). After transformation with *3×FLAG-CsWRKY22*, the expression level of *CsLOB1* was increased by approximately 2.75-fold in WT leaves but increased by only 55% in S2–5 leaves compared with that in the empty vector control (Fig. 5j). These observations suggested that the L1–2 region of the *CsLOB1* promoter is critical to the interaction between *CsLOB1* and CsWRKY22 in citrus.

## Materials and methods

### Plant materials

The citrus plant Wanjincheng orange (*C. sinensis* Osbeck) grown in an orchard of the National Citrus Germplasm Repository, Chongqing, China, was used to generate transgenic plants. All transgenic and wild-type plants were kept in a glasshouse at temperatures ranging from 25 to 30 °C in Chongqing, China.

### Vector construction

The full-length coding sequence (CDS) of *CsWRKY22* was amplified using the primers CDS-CsWRKY22-F and CDS-CsWRKY22-R by PCR from Wanjincheng orange with the incorporation of *Bgl* II and *Sal* I restriction sites. A 387-bp fragment of *CsWRKY22* was amplified using the primers RNAi-CsWRKY22-F and RNAi-CsWRKY22-R by PCR with 5′ sites for *Bam* HI and *Swa* I and 3′ sites for *Xba* I and *Asc*I. The fragment was used as an inverted repeat placed outside the intron of the chalcone synthase A gene from *Petunia hybrida* in the RNAi vector pFGC5941 (GenBank Accession No. AY310901). The *CsWRKY22* CDS and RNAi constructs were inserted into a modified binary vector pBI121-M that contained the CaMV 35S promoter and a *GUS::NPTII* fusion marker gene at the same restriction site, generating overexpression (*35S:CsWRKY22*) and RNAi (*35S:CsWRKY22* RNAi) vectors, respectively. The primers for vector construction are displayed in Table S5.

### Generation of transgenic citrus

The *35S:CsWRKY22* and *35S:CsWRKY22* RNAi constructs were transformed into *Agrobacterium tumefaciens* (EHA105) by electroporation, and then introduced into Wanjincheng orange as described previously^7^. Briefly, epicotyl explants were infected with the Agrobacterium solution and subjected to selection on a medium containing kanamycin sulfate (50 mg L^−1^). The kanamycin-resistant and GUS-positive plantlets were transferred to glasshouses and were further confirmed by PCR analysis (Novoprotein, China) for the presence of the transgenes. To avoid interference from the endogenous target gene, one end of the amplification primer was derived from the exogenous gene. Primers OE-F (tcttcgtcaacatggtggagcacga) and OE-R (ctcccggatctgcactgcttcg) were used for the overexpressing plants, and primers RNAi-F (tccaaggagctattatcggtg) and RNAi-R (cttacttacacttgccttggag) were used for the RNAi plants. The expression levels of *CsWRKY22* in transgenic plants were determined by quantitative real-time PCR (qPCR).

### RNA extraction and quantitative real-time PCR

Total RNA of citrus leaves was extracted using an EASY Spin Plant RNA Kit according to the manufacturer’s instructions (Aidlab, China). cDNA was synthesized using 0.5–1.0 µg extracted RNA with a RevertAid First-Strand cDNA Synthesis Kit (Fermentas, Canada). Then, qPCR was carried out using iTaq^TM^ Universal SYBR Green Supermix (Bio-Rad, USA) in a CFX96^TM^ Real-Time System. A previously described thermal cycling program was used^35^. Data were collected and analyzed with CFX Manager 3.1 software (Bio-Rad). qPCRs were performed in triplicate from three independent biological replicates of each sample. *Actin* was used as the internal control. The gene-specific primers used in the qPCR are displayed in Table S5.

### Evaluation of resistance to citrus canker

Healthy 6-month-old leaves from transgenic and WT plants were used to perform in vitro inoculation with our previously described methods^23^. The pathogen Xcc was isolated from naturally infected leaves of sweet orange in an orchard in Yunnan Province, China^11^. The Xcc suspension solution was introduced into the leaves either by the infiltration method (the solution was injected into the abaxial surfaces of leaves with 5-mL needleless syringes) or by the pinprick-inoculation method (1 µL suspension solution was placed into puncture holes made with a pin). Treated leaves were placed in an incubator with temperature, humidity and photoperiod settings of 28 °C, 80% and 16-h/8-h light/dark, respectively. After 10 days, the results were recorded using a digital camera. The disease severity was determined by determining the disease rate (number of diseased leaves divided by the total inoculated leaves). The disease area (cm^2^) and the growth status of Xcc were measured with previously described methods^36^.

### Microscopic analysis

Sample 10-month-old leaves were collected, cut into 2 mm × 4 mm pieces and immediately placed into a 1.5 mL centrifuge tube containing 2.5% glutaraldehyde–4% paraformaldehyde fixing solution^37^. The tissues were vacuumed to bring them into full contact with the fixing solution and then held at 4 °C overnight. The samples were rinsed three times (20 min each) with 0.05 M phosphate buffer (pH 6.9) and then dehydrated with a graded ethanol series (30, 50, 70, 95, and 100%) three times for 20 min each^38^. The dehydrated samples were infiltrated with acetone and Spurr’s resin (HEAD Biotechnology, China) at 1:3, 1:1, and 3:1 ratios overnight and in 100% fresh resin for another 24 h. Resin-infiltrated samples were vacuumed to remove any bubbles and then held at 65 °C for 20 h. Blocks were trimmed manually under a stereomicroscope with a sharp stainless steel blade. Semithin sections (800 nm) were cut with glass knives using a Leica microtome (HEAD Biotechnology, China). Sections were collected, placed in a drop of water on microscope slides, and then dried and stained with 1% toluidine blue on a hot plate at 65 °C. After a brief rinse with distilled water, the slides were examined under a light microscope equipped with a DP70 digital camera (Olympus, Japan). The leaf thickness and cell size were quantified using ImageJ software.

### RNA-Seq and analysis

Leaves were sampled from one-year-old WT and transgenic plants to perform RNA sequencing (RNA-Seq) experiments with three biological replicates. Total RNA of fully matured leaves was extracted as described above (RNA extraction and quantitative real**-**time PCR). The construction of sequencing libraries was carried out as described in a previous study^39^. The libraries were sequenced on the Illumina HiSeq 2500 platform (Majorbio, Beijing, China). All clean reads were aligned to the sweet orange genome (http://citrus.hzau.edu.cn/orange/index.php) with HISAT 2.0.5 software after filtering^40^. The Nr, Nt, Pfam, KOG/COG, Swiss-Prot, KO, and GO databases were used to annotate gene ontology. Gene expression levels were indicated by the FPKM (fragments per kilobase of exon per million fragments mapped) values^41^. The DESeq2 R package (1.10.1) was used to identify differentially expressed genes (DEGs) according to the following thresholds: fold change ≥2 and adjusted *P*-value < 0.05. The DEG lists were used for KEGG pathway and GO term analyses using the web-based tools KOBAS 3.0^42^ and agriGO v2.0^43^, respectively. The RNA-seq data were deposited in the NCBI SRA with accession number PRJNA667275.

### Subcellular localization analysis

The CDS of *CsWRKY22* without the stop codon was amplified using the primers CsWRKY22-GFP-F and CsWRKY22-GFP-R with *Kpn* I and *Sal* I restriction sites and inserted into a *35S:GFP* expression vector to generate a *35S:CsWRKY22-GFP* cassette. The fusion construct and the control vector (*35S:GFP*) were introduced into *A. tumefaciens* (EHA105) and agroinfiltrated into tobacco (*Nicotiana benthamiana*) leaves for transient expression, respectively^44^. The treated plants were cultured for three days, and then DAPI (4′-6-diamidino-2-phenylindole) was used to stain the nuclei^45^. The fluorescence was examined by confocal microscopy (Olympus, Japan).

### Transcriptional activation activity assay

The full-length CDS of *CsWRKY22* was amplified by PCR using the primers pGBKT7-CsWRKY22-F and pGBKT7-CsWRKY22-R and was ligated downstream to the GAL4 DNA binding domain (GAL4BD) in the pGBKT7 vector (Clontech, USA) by homologous recombination. The fusion construct and empty vector control were transformed into the yeast strain Y2HGold and plated on synthetic dropout medium without tryptophan (SDO). Then, the transformed cells were cultured on selective SDO medium supplemented with 40 μg/mL X-α-gal (5-bromo-4-chloro-3-indolyl-α-d-galactoside) and 200 ng/mL AbA (aureobasidin A) (SDO/X/A) to assess the transcriptional activation potential of CsWRKY22 for the *MEL1* (encodes α-galactosidase) and *AUR1* (encodes the enzyme inositol phosphoryl ceramide synthase) reporter genes.

### Dual LUC assays

The *CsLOB1* promoter (a 500 bp fragment upstream of the start codon)^7^ was amplified by PCR using the primers pGreen-pLOB1-F and pGreen-pLOB1-R and was inserted ahead of the LUC gene in the pGreenII 0800 vector to form a promoter-LUC reporter construct. The full-length CDS of *CsWRKY22* was fused downstream to the CaMV35S promoter in a modified binary vector pBI121-M to generate an effector construct. The construct and an empty control vector were introduced into *A. tumefaciens* (GV3101) and agroinfiltrated into tobacco (*N. benthamiana)* leaves for the transient gene expression assay. The Dual-Glo® Luciferase Assay System (Promega) was used to examine the firefly and Renilla luminescence according to the manufacturer’s instructions.

### Yeast one-hybrid (Y1H) assay

The *CsLOB1* promoter (L1) was amplified by PCR and dissected into two different subfragments (L1-1, −349 to −254 bp; L1-2, −188 to −153 bp) according to the location of the W-box elements (TGAC). The Y1H assay was carried out according to the methods in a previous study^46^. All three promoter fragments were then individually inserted into the pLacZi vector as baits, and the full-length CDS of *CsWRKY22* was fused with the activation domain in the pB42AD vector to construct the prey. Plasmids were cotransformed into the yeast strain EGY48. Protein–DNA interactions were determined based on the blue color of cotransformed yeast cells on synthetic dropout medium without tryptophan and uracil (SD/-Trp/-Ura) plates supplemented with X-gal (5-bromo-4-chloro-3-indolyl β-d-galactoside). pB42AD/pLacZi-L1, pB42AD/pLacZi-L1-1, and pB42AD/pLacZi-L1-2 were used as negative controls. The mutated (TCAA) W-box was used to generate baits to determine the specific binding of CsWRKY22 to the W-box region.

### Electrophoretic mobility shift assay (EMSA)

The CDS of *CsWRKY22* was cloned into the prokaryotic expression vector pGEX-4T-1, which contains a GST tag, and was expressed in the *Escherichia coli* strain BL21 (DE3). The expression and purification of the GST-CsWRKY22 protein were performed as described in previous studies^47^. A single-stranded oligonucleotide was synthesized based on sequences of the wild-type probe (5′-attaatgacatcatctagtggctcggtgacatacgc-3′) or mutated probe (5′- attaatgaaatcatctagtggctcggtgtcatacgc-3′) and labeled with biotin by Shanghai Sangon Biotechnology (Shanghai, China); the original fragment without biotin labeling was used as a competitor. EMSA was performed using the LightShift Chemiluminescent EMSA Kit according to the manufacturer’s instructions (Thermo Scientific, USA).

### Transient gene expression analysis in citrus

The 3×FLAG-CsWRKY22 fusion protein was under the control of the CaMV35S promoter in a modified binary vector, pBI121-M. The construct was transformed into the *A. tumefaciens* strain GV3101 and transiently introduced into the leaves of the WT and *CsLOB1* promoter mutant S2-5^7^. *Agrobacterium*-mediated transient expression in citrus leaves was performed as described in previous studies with some modifications^48^. Well-expanded, light green, young citrus leaves were collected and cleaned with 75% alcohol for 30 s and then washed three times with sterile water. A 0.5 mm needle was used to wound the abaxial surface of the leaves. Then, the Agrobacterium suspension was infiltrated into the leaves using a 10 mL needleless syringe. The leaves were incubated in darkness at 28 °C for 6 days.

### Statistical analysis

Statistical analysis of the data was performed with SPSS V20 software. The averages ± standard deviations (SDs) were adopted to indicate the measured values. The significance of the differences was tested with Duncan’s multiple range test for ANOVA (analysis of variance) (*P* < 0.05, different letters indicate significant differences) or with a two-tailed Student’s *t*-test (*P < 0.05; **P < 0.01).

## Discussion

### CsWRKY22 promotes cell enlargement

Cell enlargement in plants requires many coordinated processes, including cell wall synthesis and expansion^49^. Cell wall remodeling is a prerequisite for cell enlargement^50^. Expansins, β-1,4-endoglucanase, pectin lyase, and cellulose synthase contribute to cell wall organization and biogenesis, which are involved in cell wall expansion and loosening processes^24–29^. The auxin-inducible genes *SAUR* (auxin-induced protein), *GH3* (indole-3-acetic acid-amido synthetase), and *IAA* (auxin-responsive protein) play vital roles in regulating cell wall loosening^30,31,51^. In addition, brassinosteroids (BRs) are known to promote cell enlargement by increasing the activities of the BRU1 and TCH4 genes, that encode the xyloglucan endotransglycosylase proteins responsible for cell wall relaxation^32,33^. In this study, we observed that the spongy mesophyll cells of *CsWRKY22*-overexpressing plants were enlarged compared with those of the WT control (Fig. 2). Through transcriptome analysis, we found that the overexpression of CsWRKY22 enhanced the expression of expansin, β-1,4-endoglucanase, pectin lyase, and cellulose synthase genes. Furthermore, auxin-inducible genes and BR-regulated xyloglucan endotransglucosylase/hydrolase genes were also induced in *CsWRKY22*-overexpressing plants (Fig. 4); this finding supports the idea that CsWRKY22 could promote cell enlargement through the activation of cell wall organization or biogenesis as well as the auxin and BR hormonal signaling pathways in citrus. Interestingly, Kloth et al.^18^ showed that cell wall loosening-related genes, such as pectin lyases, expansins and the auxin hormonal signaling pathway, were downregulated in aphid-infested *wrky22* knockout Arabidopsis. These findings imply that WRKY22 members can act as a general regulating factor for cell growth in plants.

### CsWRKY22 directly regulates the expression of *CsLOB1*

*CsLOB1* is a crucial disease susceptibility gene that is involved in the development of citrus canker; it can be directly regulated by the effector PthA4, which is secreted by Xcc^11^. The mutation of the *CsLOB1-*encoding region or the binding site of effector PthA4 in the promoter by CRISPR gene-editing technology resulted in enhanced citrus canker resistance^7,52^. However, Xcc strains with missing PthA4 could still induce the expression of *CsLOB1*, suggesting that Xcc can regulate the expression of *CsLOB1* through other mechanisms^12^.

The expression of *CsLOB1* is related to the expression of genes associated with cell expansion^7,11^. Duan et al. indicated that 12 genes associated with cell wall loosening and cell growth, including expansin, pectate lyase, endoglucanase, polygalacturonase, and gibberellin-regulated genes, were induced by CsLOB1 and then promoted pustule formation^47^. This evidence indicates that both CsWRKY22 and CsLOB1 participate in regulating cell growth. At the same time, we found that the overexpression of *CsWRKY22* caused dwarf phenotypes similar to the phenotypes observed in transgenic apple, *Arabidopsis*, and *Populus* overexpressing *LBD* genes^53,54^; this finding indicates that there are intersections between the functions of the two genes in the regulation of plant development. The transcriptome and qPCR results showed that the expression of *CsLOB1* was upregulated in the *CsWRKY22*-overexpressing plants (Fig. 5a and Table S2). The tobacco and citrus transient expression assays clearly confirmed that *CsWRKY22* upregulated *CsLOB1* expression by activating the *CsLOB1* promoter. All the data reveal that *CsWRKY22* can function upstream of *CsLOB1* to regulate citrus plant development.

Transcription factors can modify the expression potential of target genes by recognizing the regulatory DNA motifs of target genes^55^. WRKY transcription factors specifically recognize and bind to a DNA cis-acting element [(C/T) TGAC (C/T)] called a W-box^56^. It is generally believed that promoter sequences whose core sequence is TGAC have certain W-box functions^57^. It has been shown that when any nucleotide of TGAC in the core sequence of the W-box is replaced, the binding ability of the WRKY transcription factor to the W-box will be greatly reduced or will completely disappear, indicating that this core sequence is necessary for the binding of WRKY proteins. Therefore, the W-box can be used to identify and screen for target genes of the WRKY transcription factor^58^. In our study, we found that the promoter region of *CsLOB1* contained four W-boxes (Fig. S4). We further used dual LUC assays, Y1H, EMSA, and transient expression analysis to confirm that CsWRKY22 could activate the expression of *CsLOB1* by directly binding to the W-boxes near the transcription start site of *CsLOB1* in vivo and in vitro (Fig. 5). The identification of the CsWRKY22–*CsLOB1* interaction deepens our understanding of the regulation of *CsLOB1* expression and of canker susceptibility in citrus.

### CsWRKY22 increases susceptibility to citrus canker

WRKY22 plays a role downstream of the MAPK pathway, participates in the basic immune response of plants, and is a marker gene of plant PTI^17^. In Arabidopsis, T-DNA insertion mutants of *wrky22* are more susceptible to *Pseudomonas syringae* than WT^20^. In rice, *wrky22* mutants show reduced resistance to *Magnaporthe oryzae*, whereas the overexpression of *OsWRKY22* results in enhanced disease resistance^19^. In pepper, CaWRKY22 contributes to resistance to *Ralstonia solanacearum* by orchestrating networks with CaWRKY6, CaWRKY27, CaWRKY40, and CaWRKY58^21^. These results indicate that WRKY22 genes act as positive regulators of resistance to bacterial pathogens in these plants. In contrast, AtWRKY22 can promote the susceptibility of Arabidopsis to green peach aphids (*Myzus persicae*) by suppressing the SA and JA signaling pathways^18^. In our previous study, the CRISPR/Cas9‐mediated editing of *CsWRKY22* reduced susceptibility to Xcc in Wanjincheng orange^22^. In this study, the excessive expression of *CsWRKY22* increased susceptibility to citrus canker, while RNAi interference with *CsWRKY22* increased resistance to citrus canker in Wanjincheng orange (Fig. 3). Our accumulating evidence shows that *CsWRKY22* is a susceptibility gene for citrus canker. The above studies suggest that WRKY22 members play different roles in the responses to different biotic stresses in different plants.

*CsWRKY22* can be triggered by the Xcc‐derived PAMP flagellin (Xflg22), which is an elicitor of the MAPK cascade, in “Nagami” kumquat (*Fortunella margarita*)^59,60^. In this study, we found that CsWRKY22 is not solely involved in PTI but also participates in cell growth. In the KEGG pathway enrichment analyses of the differentially expressed genes (DEGs), we indeed found that two innate immunity response-related pathways, MAPK signaling (FDR = 0.194) and plant–pathogen interaction (FDR = 0.252), were identified (Table S4). Although the expression of the LRR receptor-like serine/threonine-protein kinase gene *FLS2* (Cs1g14210), which is involved in triggering the innate immune response, was downregulated, the majority of genes enriched in these two pathways were upregulated, including pathogenesis-related protein 1-like (Cs8g03430) and respiratory burst oxidase homolog protein (Cs8g12000) (Table S6 and S7). These results suggested that the innate immune response was enhanced in *CsWRKY22*-overexpressing citrus, which was consistent with the positive role of WRKY22 in pathogen defense in other plant species^19–21^. However, these two immunity-related pathways were not as significant as the plant hormone signal transduction pathway (FDR = 0.000868), which contributes to cell enlargement and host susceptibility^3^ (Table S4). We noted that the Cs5g25070 gene, which was predicted to encode a cytochrome P450, CYP83B1, had the highest level of expression in the *CsWRKY22*-overexpressing citrus (Table 1). CYP83B1 metabolizes the tryptophan-derived aldoxime intermediate “indole-3-acetaldoxime” that serves as a precursor for auxin biosynthesis in Arabidopsis^58^. Thus, CsWRKY22 could positively affect citrus canker susceptibility through its regulation of CYP83B1 activity in auxin biosynthesis. Combined with the upregulation of the key susceptibility gene *CsLOB1*, our results suggest that the negative effects of CsWRKY22 overwhelmed its positive effects in the regulation of citrus resistance to Xcc, which ultimately led to the increased susceptibility of the *CsWRKY22*-overexpressing citrus to the Xcc pathogen.

**Fig. 6:**
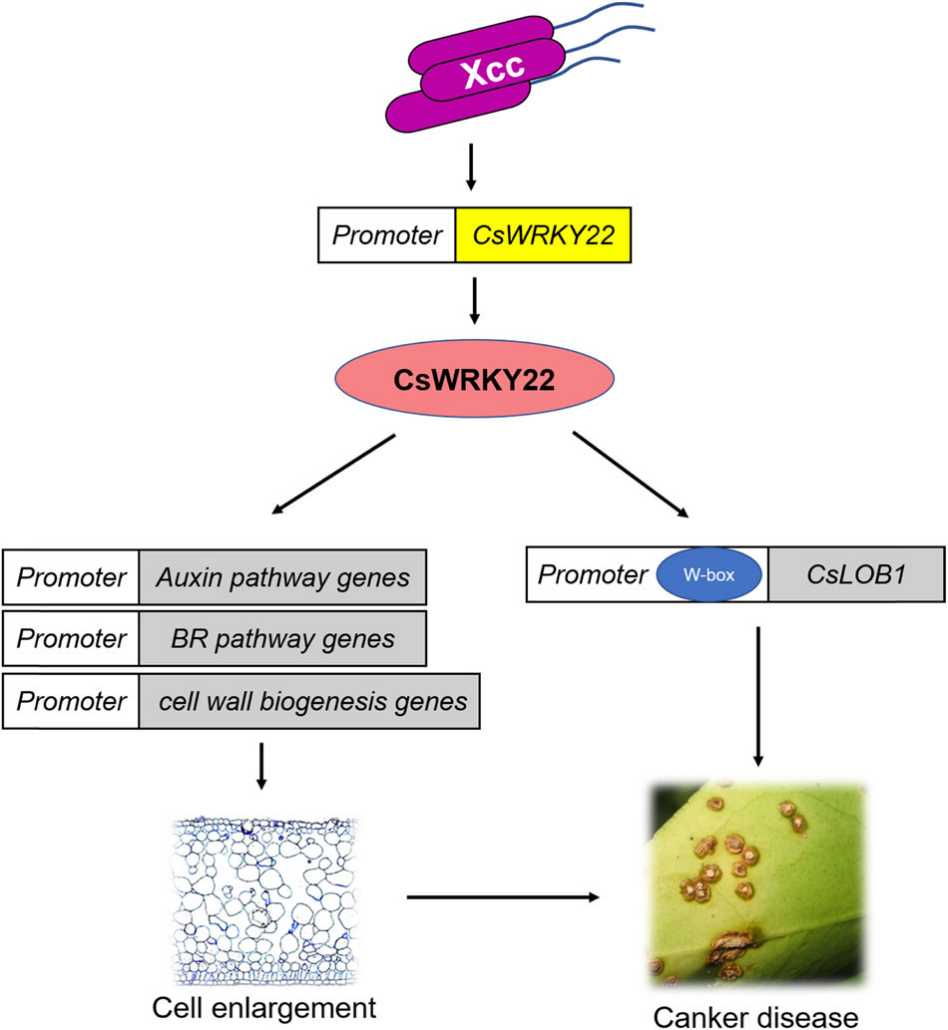
Hypothetical model of CsWRKY22’s role in the development of citrus canker. Xcc infection results in the upregulation of CsWRKY22 expression. CsWRKY22 promotes the expression of genes in the auxin and BR signaling and cell wall remodeling pathways and the expression of the citrus canker susceptibility gene *CsLOB1*. These changes contribute to cell enlargement and canker disease

Collectively, we conclude that CsWRKY22 increases the susceptibility of Wanjincheng orange to canker disease through the induction of cell enlargement and the activation of *CsLOB1* expression in (Fig. 6). The discovery of the divergent biological functions and molecular mechanisms of WRKY22 members will help us to manipulate host plant defenses for resistance breeding using WRKY22 genes.

## Supplementary information

Fig. S1-4, Table S1 & Table S5

Table S2 Differentially expressed genes in transgenic lines

Table S3 GO analysis of the differentially expressed genes

Table S4 KEGG pathways enriched among the DEGs

Table S6 Differentially expressed genes in MAPK signaling pathway

Table S7 Differentially expressed genes in plant-pathogen interaction
