# Supplementary material for: Transcription factor WRKY22 regulates canker susceptibility in sweet orange (Citrus sinensis Osbeck) by enhancing cell enlargement and CsLOB1 expression
Source: Hortic Res. 2021 Mar 1;8:50. doi: 10.1038/s41438-021-00486-2 (PMC7917094; doi:10.1038/s41438-021-00486-2)
Supplement: Supplementary file 1 — Fig. S1-4, Table S1 & Table S5 [file 41438_2021_486_MOESM1_ESM.docx]

**
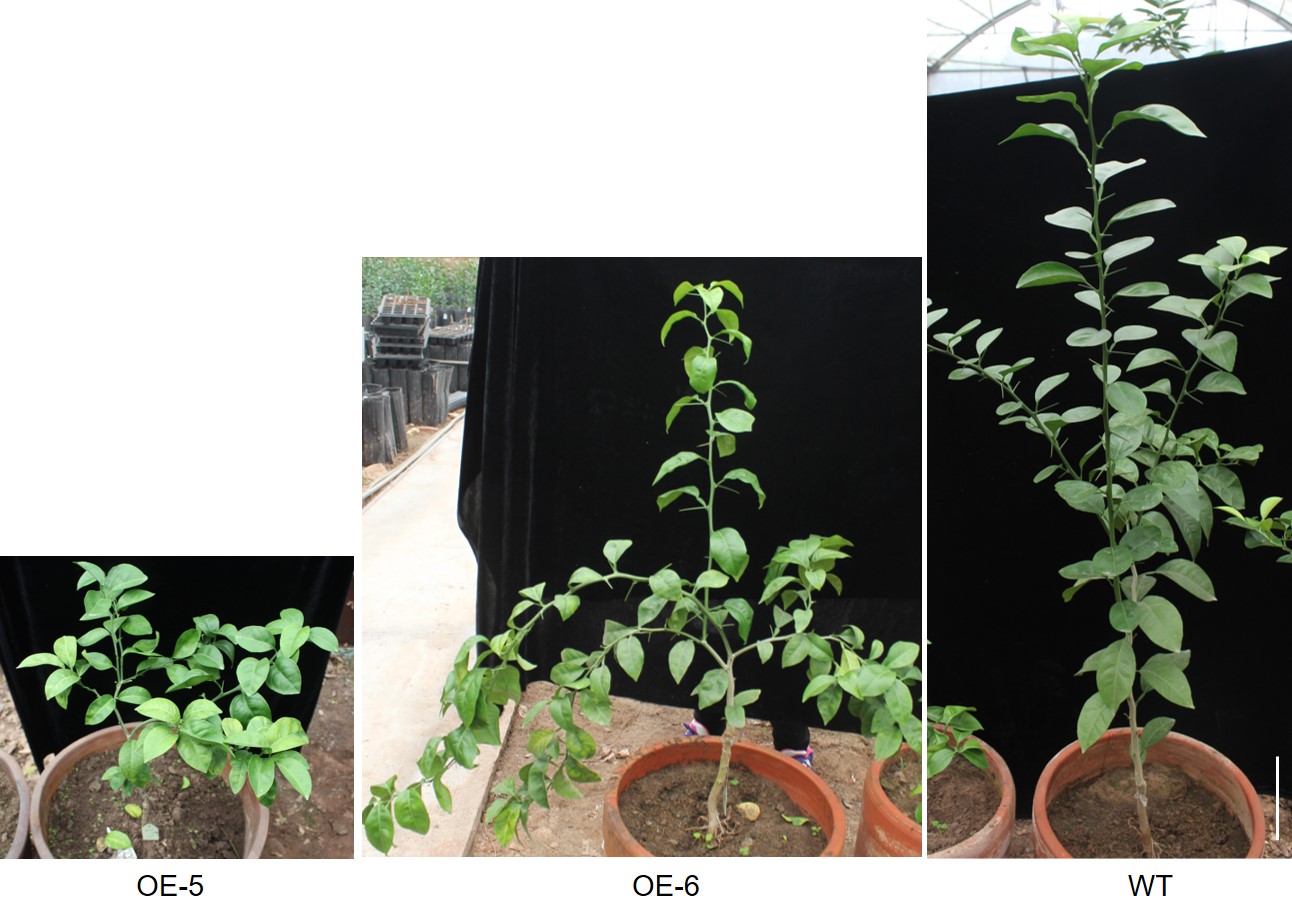
**

Fig. S1 Phenotype characteristics of three years old *CsWRKY22* over-expressing plants. Bar = 15 cm.


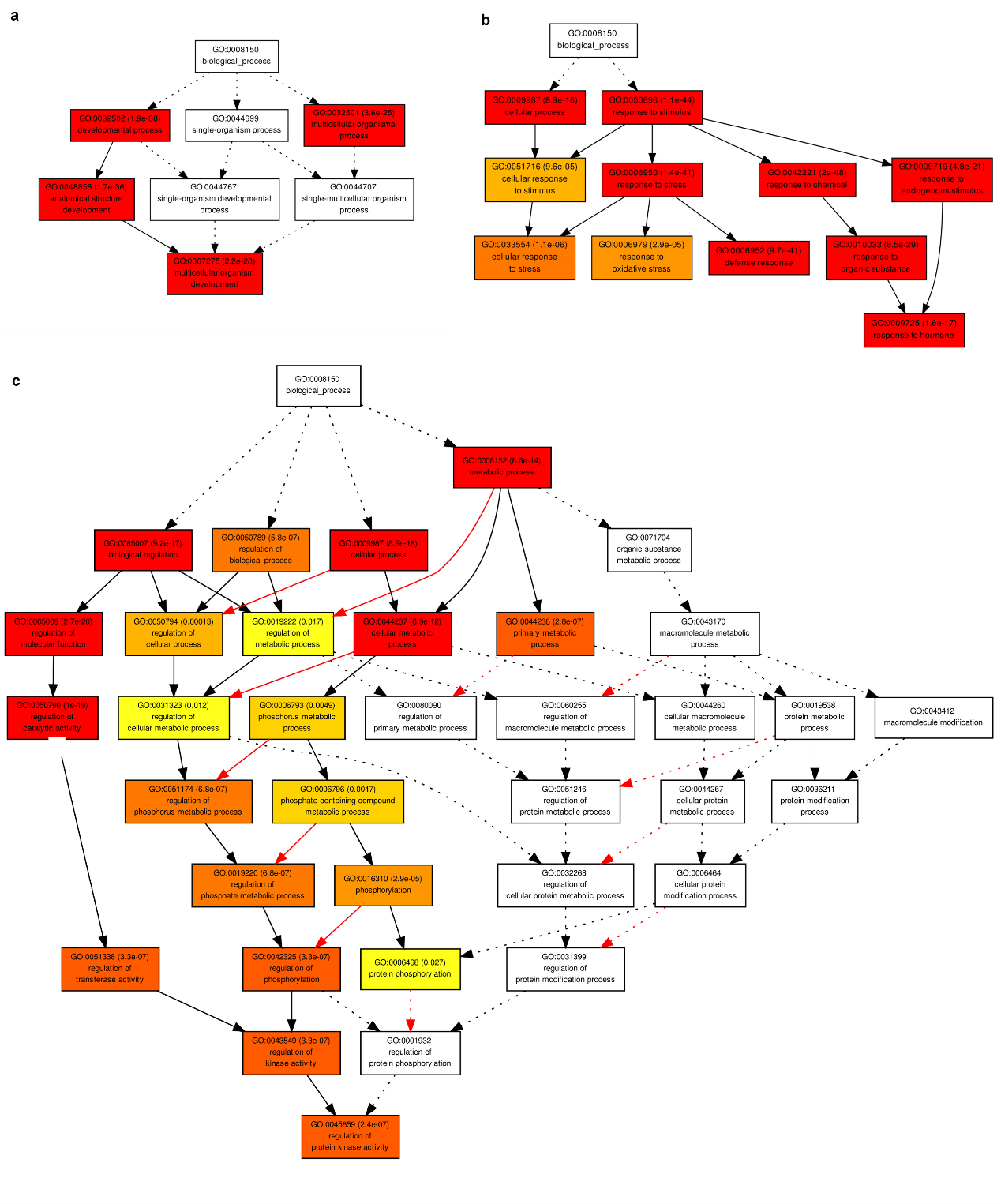


Fig. S2 Thumbnails view of directed acyclic graphs (DAGs) on development (a), response to stimulus (b), and kinase (c).


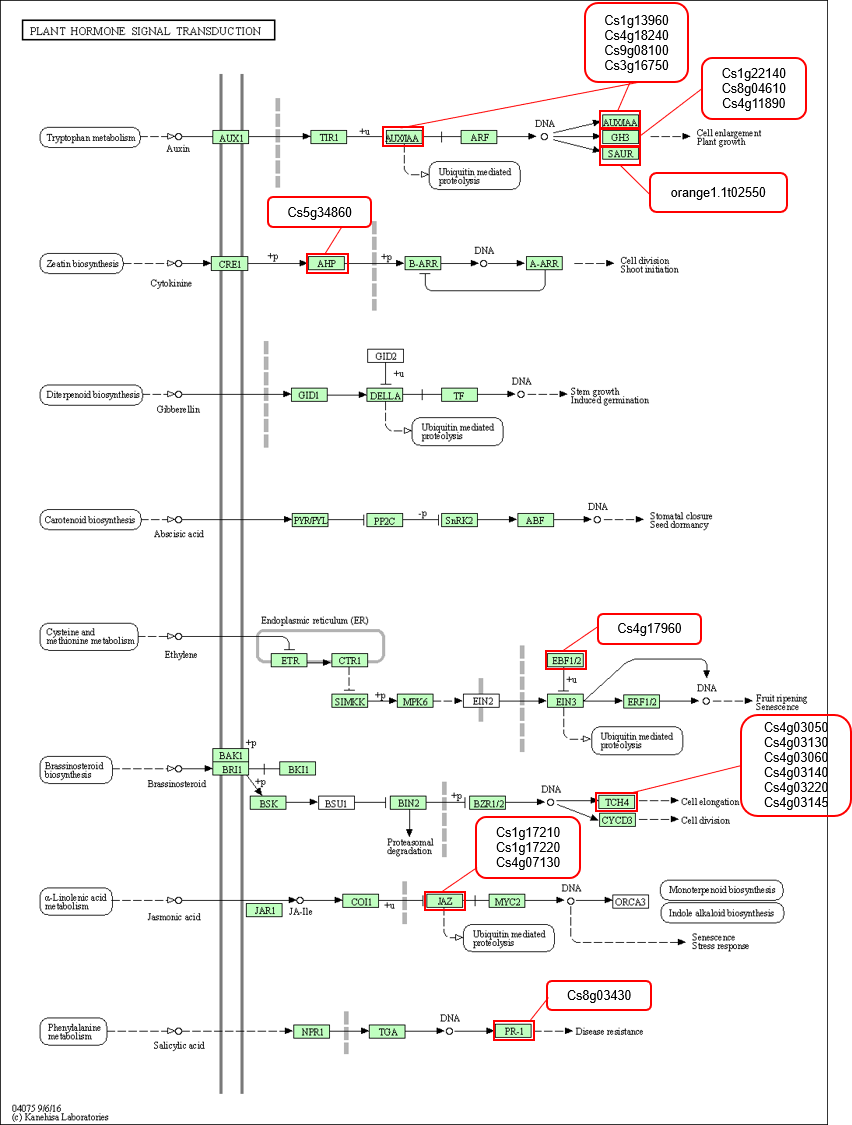
Fig. S3 KEGG mappings of plant hormone signal transduction pathway. Red indicates up-regulated genes.


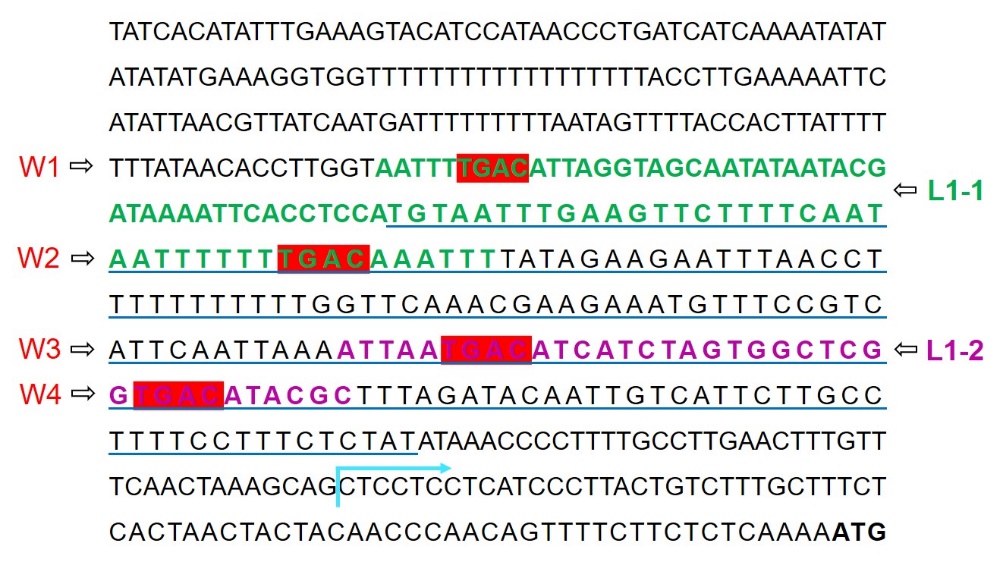


Fig. S4 Characterization of the *CsLOB1* promoter sequence reported by Peng et al.^1^. Red-Backed letters show potential CsWRKY22 binding sites (W-boxes; W1, W2, W3, W4). Green letters show the L1-1 fragment. Purple letters show the L1-2 fragment. The underlined area was the deletion of S2-5 line reported by Peng et al.^1^. Blue arrow indicates the predicted transcriptional initiation site^2^. ATG is the initiation codon of *CsLOB1*.

**Table S1 Summary of sequencing data for each sample**

| Sample | Raw reads Number | Raw Data Size (bp) | Clean reads Number | Clean Data Size (bp) | Clean Data Rate (%) |
| --- | --- | --- | --- | --- | --- |
| WT_1 | 49413472 | 7410000000 | 49085866 | 7280000000 | 99.98 |
| WT_2 | 47352088 | 7100000000 | 47076764 | 7000000000 | 99.98 |
| WT_3 | 56163006 | 8420000000 | 55853092 | 8310000000 | 99.98 |
| OE-5_1 | 56281084 | 8440000000 | 55771246 | 8260000000 | 99.98 |
| OE-5_2 | 64810058 | 9720000000 | 64285228 | 9540000000 | 99.98 |
| OE-5_3 | 57870096 | 8680000000 | 57427282 | 8510000000 | 99.98 |
| OE-6_1 | 57852982 | 8677947300 | 57362908 | 8512300937 | 99.99 |
| OE-6_2 | 55371036 | 8305655400 | 54856000 | 8128666153 | 99.98 |
| OE-6_3 | 52831344 | 7924701600 | 52509460 | 7807986706 | 99.98 |

WT, wild type; OE-5 and OE-6, *CsWRKY22* over-expressing lines.

**Table S5 Primers used in this study**

| Primer name | Primer sequence (5' to 3') |
| --- | --- |
| CDS-CsWRKY22-F | GCCAGATCTATGGACTGGGATTTGCAAGC |
| CDS-CsWRKY22-R | TTCGTCGACTCAGAGACCACCGTTCAGGG |
| RNAi-CsWRKY22-F | GCTCTAGAGGCGCGCCCCTTATCCAAGGAGCTATTATC |
| RNAi-CsWRKY22-R | CGCGGATCCATTTAAATCCCTTCCAAATCCTCCAAGC |
| CsWRKY22-GFP-F | CGGGGTACCATGGACTGGGATTTGCAAGC |
| CsWRKY22-GFP-R | TTCGTCGACGAGACCACCGTTCAGGGTAAC |
| pGBKT7-CsWRKY22-F | CATGGAGGCCGAATTCATGGACTGGGATTTGCAAGCCATAG |
| pGBKT7-CsWRKY22-R | GCAGGTCGACGGATCCTCAGAGACCACCGTTCAGGGTAAC |
| pGreen-pLOB1-F | CCCAAGCTTCACATATTTGAAAGTACATC |
| pGreen-pLOB1-R | CGGGATCCTTGAGAGAAGAAAACTGTTGGG |
| RT-CsActin-F | CTGCCTGATGGCCAGATCAT |
| RT-CsActin-R | TACCAGCAGCTTCCATTCCG |
| RT-CsWRKY22-F | TCCAAGGAGCTATTATCGGTG |
| RT-CsWRKY22-R | CTCCCGGATCTGCACTGCTTCG |
| RT-3×FLAG-CsWRKY22-F | GATGATGATGATAAGATGGACTGG |
| RT-3×FLAG-CsWRKY22-R | CTCACTGCTGCAACCTCTTACTAT |
| RT-CsLOB1-F | CTGCCAGAATCTCAACGAGC |
| RT-CsLOB1-R | TTGGCTAACTGAGCCTGAAGC |

1. Peng, A.H. et al. Engineering canker-resistant plants through CRISPR/Cas9-targeted editing of the susceptibility gene *CsLOB1* promoter in citrus. *Plant biotech. J.* **15**, 1509–1519 (2017).

2. Xu, Q. et al. The draft genome of sweet orange (*Citrus sinensis*). *Nat. genet.* **45**, 59-66 (2013).
